# Non-native PGPB Consortium Altered the Rhizobacterial Community and Slightly Stimulated the Growth of Winter Oilseed Rape (*Brassica napus* L.) Under Field Conditions

**DOI:** 10.1007/s00248-024-02471-3

**Published:** 2025-01-08

**Authors:** J. Dobrzyński, I. Kulkova, Z. Jakubowska, B. Wróbel

**Affiliations:** Institute of Technology and Life Sciences—National Research Institute, Falenty, 3 Hrabska Avenue, 05-090 Raszyn, Poland

**Keywords:** Plant growth-promoting bacteria, Native microbiota, Diversity of bacteria, Eco-friendly biostimulator

## Abstract

Plant growth-promoting bacteria (PGPB) are among the most promising alternatives to mineral fertilizers. However, little is known about the effects of applied bacteria on the native microbiota, including the rhizobacterial community, which plays a crucial role in bacteria-plant interactions. Therefore, this study is aimed at assessing the effects of PGPB not only on plants but also, importantly, on the native rhizobacterial community of winter oilseed rape. The bacterial consortium, consisting of *Pseudomonas* sp. KR227 and *Azotobacte*r PBC1 (P2A), slightly promoted plant growth, increasing the root weight by 21.95% and seed yield by 18.94%. This likely results from its ability to produce indole-3-acetic acid (IAA), solubilize phosphorus, and fix nitrogen, as indicated by a 35.76% increase in N-NH_4_ and a 35.05% increase in available phosphorus (AP). The introduced PGPB altered the rhizobacterial community of rapeseed, increasing the relative abundance of the phylum Proteobacteria and the genus *Pseudomonas* while decreasing the relative abundance of phylum Verrucomicrobiota (3 weeks after inoculation). Moreover, Proteobacteria were positively correlated with AP, while Verrucomicrobiota were correlated with N-NH_4_. At the genus level, *Flavobacterium* and *Pseudomona*s were positively correlated with AP, whereas *Candidatus* Udaeobacter showed a positive correlation with N-NH_4_ and a negative correlation with pH. Importantly, the P2A consortium did not significantly affect the diversity of native rapeseed rhizobacteria. These findings suggest that the tested P2A consortium has potential as a biostimulant in rapeseed cultivation.

**Supplementary Information:**

The online version contains supplementary material available at 10.1007/s00248-024-02471-3.

## Introduction

Oilseed rape (*Brassica napus* L.) is one of the most important oil crops, ranking behind only soybean and palm oil. In recent decades, there has been a continuous increase in the farming area under oilseed rape (FAO report). According to the FAO report [1], the global area dedicated to oilseed rape in 2023 reached 33.7 million hectares, with yields totaling approximately 68.9 million metric tonnes. The regions that are the largest oilseed rape producers include China (16.32 mln ton), Canada (18.8 mln ton), the European Union (19.3 mln ton), Australia (5.7 mln ton), and the USA (1.9 mln ton). In the European Union (0.75%), the largest areas of oilseed rape are located in France (4.2 mln ton) and Germany (4.2 mln ton), with Poland in 10th place [1–3]. However, oilseed seed production requires relatively more inputs, compared to other crops, particularly involving the use of fertilizers such as nitrogen [4, 5]. Therefore, long-term cultivation of crops can have detrimental effects on the environment, including soil acidification, loss of biological diversity, and eutrophication of streams and water reservoirs [6–8]. An eco-friendly alternative to reduce the use of mineral fertilizers in oilseed rape cultivation is the application of plant growth-promoting bacteria (PGPB) [9, 10]. These bacteria stimulate plant growth by producing various biologically active substances, such as phytohormones (IAA, gibberellins, cytokinins) and enzymes (ACC deaminase and enzymes involved in the decomposition of biomass: cellulases, ligninases, chitinases), as well as by fixing atmospheric nitrogen (nitrogenase) and solubilizing forms of phosphorus unavailable to plants [11, 12]. The use of PGPB aligns with the EU’s agricultural objectives, including the European Green Deal (EGD) and the EU Biodiversity Strategy for 2030, which emphasize biodiversity protection and promotion of biological fertilizers [13]. However, even biological solutions require thorough investigation before commercialization, particularly regarding their effects on the native soil microbiota, including, for example, the diversity and taxonomic composition of the bacterial community [14, 15]. It should be noted that, to date, only a few studies have reported on the response of the native bacterial community to PGPB applications in oilseed rape cultivation [16–18]. However, to the best of our knowledge, no studies have assessed the effects of PGPB on the rhizobacterial community of winter oilseed rape under field conditions using Next-Generation Sequencing (NGS).

Thus, the study is aimed not only at investigating the response of plants and soil chemical properties to the introduction of PGPB but primarily at assessing the reaction of the bacterial community in the rhizosphere, where plant-bacteria interactions occur, during various stages of oilseed rape cultivation under field conditions. Specifically, the study examines the diversity of bacteria (both alpha and beta diversity) and the taxonomic composition of the rhizosphere bacterial community following the inoculation of a rapeseed crop with a consortium of *Pseudomonas* sp. KR227 and *Azotobacter* sp. PBC 1.

## Methods

### PGPB Isolation, Selection, and Taxonomic Identification

In the study, approximately 200 bacterial strains were isolated from various soil samples, including both rhizosphere and bulk soil. Initially, 10 g of roots or bulk soil was suspended in 100 mL of sterile NaCl solution (0.85%) and shaken at 200 rpm. Then, the resulting suspension was diluted to a concentration of 10^−5^ or 10^−6^ and, in a petri dish, 1 mL of the diluted suspension was inoculated with nutrient agar. Colonies were then selected and repeatedly passaged until pure bacterial strains were obtained. Next, the selected isolates were tested for mutual antagonism using the plate method (cross-streak method).

Bacterial strain identification was based on 16S rRNA gene sequencing. Genomic DNA from the bacterial strains, cultivated for 16 h, was extracted using the Genomic Mini kit (A&A Biotechnology). To amplify the 16S rRNA genes, universal primers 27F (5′-AGAGTTTGATCCTGGCTCAG-3′) and 1492R (5′-GGTTACCTTGTTACGACTT-3′) were used, as described by Kim et al. [19]. PCR amplification was performed under the following conditions: initial denaturation at 95 °C for 3 min, followed by 30 cycles of denaturation at 95 °C for 30 s, annealing at 55 °C for 2 min, extension at 72 °C for 2 min, and finally incubation at 72 °C for 10 min for DNA amplification. The purified PCR products were sequenced using the Sanger technique (NEXBIO, Lublin, Poland). The forward and reverse reads were assembled into contigs using BioEdit (version 7.2) and compared with sequences from GenBank and EMBL databases via BLAST (Basic Local Alignment Search Tool). The 16S rRNA gene sequences of studied bacteria strains have been deposited in GenBank under accession numbers: *Pseudomonas* p. KR227 – PP500614 and *Azotobacter* sp. PBC1 – PP500626.

### IAA Production

IAA production was determined following the method described by Bric et al. [20]. For IAA production assessment, bacterial isolates were cultured in Luria–Bertani (LB) broth supplemented with 1000 μg L^−1^ tryptophan for 72 h at 30 ± 2 °C. The cultures were then centrifuged at 12,000 rpm m-1 for 8 min at 3 °C. The resulting supernatant (1 mL) was mixed with 2 mL of Salkowski reagent (49 mL, 35% perchloric acid, 1 mL 0.5 M FeCl_3_ solution). Absorbance was measured at 530 nm using a spectrophotometer (Epoch 2 Plate Reader, BioTek). Bacterial IAA production was quantified using a standard IAA curve, calibrated in the range of 10–100 μg mL⁻^1^.

### Nitrogen Fixation

Nitrogen fixation tests for the bacterial strains were conducted using nitrogen-free Burk’s medium which contained the following components: MgSO₄ (0.2 g), K₂HPO₄ (0.8 g), KH₂PO₄ (0.2 g), CaSO₄ (0.13 g), FeCl₃ (0.001 g), NaMoO₄ (0.0003 g), and sucrose (20 g), with agar (18 g L⁻^1^). The cultures were incubated at 30 °C for 7 days. Bacteria that grew on the medium were identified as diazotrophs [21].

### Solubilization of P and Zn

Bacterial strains were spot-transferred onto Pikovasky agar (per liter: yeast extract 0.5 g, dextrose 10.0 g, Ca_3_(PO_4_)_2_ 5.0 g, (NH_4_)_2_SO_4_ 0.5, KCl 0.2 g, MgSO_4_ 0.1 g, MnSO_4_ 0.0001 g, FeSO_4_ 0.0001 g, agar 15.0 g), supplemented with 2% tricalcium phosphate (TCP), and incubated at 30 ± 2 °C for 94 h. The ability to solubilize was assessed by the presence of clear zones around the colonies.

Zinc solubilization was tested by spot inoculating strains onto Tris-minimal medium (per liter: Tris–HCl 6.06 g, NaCl 4.68 g, KCl 1.49 g, NH_4_Cl 1.07 g, Na_2_SO_4_ 0.43 g; MgCl_2_ 2H_2_O 0.2 g; CaCl_2_ 2H_2_O, 30 mg, pH 7.0), supplemented with 2% agar and 0.1% insoluble zinc in the form of zinc sulfate (ZnSO_4_) [22]. The bacterial cultures were incubated at 30 ± 2 °C for 14 days, after which the presence of clear zones around the colonies, indicating zinc solubilization, was observed.

### Carboxymethyl Cellulase (CMCase) Activity

Cellulolytic activity (CMCase) of the isolates was assessed by spot inoculating Park’s culture medium (per liter: (NH_4_)_2_ SO_4_ 0.5 g; KH_2_PO_4_ 1.0 g; KCl 0.5 g; MgSO_4_ 0.2 g; CaCl_2_ 0.1 g), supplemented with 1% carboxymethylcellulose (CMC) solution. The plates were incubated for 120 h at 28 ± 2° and then checked for clear zones, which indicated cellulolytic activity.

### Experimental Site, Design, and Soil Sampling

The research was conducted on a field owned by the Awista Pierwsza company in Kobierzycko, Sieradz district, Łódź Voivodeship (51° 37′ 59.2″ N 18° 36′ 50.9″ E). Winter oilseed rape (*Brassica napus* L.; variety Absolut) was sown at a rate of 40 seeds per m^2^ in August 2022 on Luvisol clay. In autumn 2022, the field was fertilized with Macro Speed Optima (16 P, 32 K, 11 Ca, 8 SO_3_, 8 Zn) at a rate of 200 kg ha^−1^ and urea with a urease inhibitor (46 N) at a rate of 180 kg ha^−1^. At the start of the growing season, the average soil pH was 6.1. During the experimental period, the average temperature and precipitation were as follows: April—24.38 [mm]; 7.8 [°C], May—19.05 [mm]; 13.2 [°C], June—30.47 [mm]; 18.20 [°C], July—85.1 [mm]; 20.0 [°C]. In spring 2023, a one-factor field experiment was established on a production field using a randomized block design in triplicate. Each plot measured 2 m × 5 m (10 m^2^), with a meter-wide buffer zone between plots. The experiment included two treatments: control (C) and a bacterial consortium consisting of *Pseudomonas* sp. KR227 and *Azotobacter* sp. PBC1 (P2A). The bacterial application procedure was carried out on 18 April 2023 (BBCH 35), using a hand sprayer from approximately 50 cm above the soil, with 600 mL of inoculum (1.5 × 10⁸ cells mL⁻^1^) applied per plot. Roots (rhizosphere soil) were sampled three times during the growing season: 3 weeks after the application of the consortium (oilseed rape development – BBCH 65 oilseed rape development stage), at mid-season (oilseed rape development stage–BBCH 75 oilseed rape development stage), and at harvest (BBCH 90). Roots from 3 plants per plot were taken for study; samples were taken in triplicate (3 replicates from each treatment). To obtain the rhizosphere, loosely bound soil was shaken off, and tightly bound soil was extracted with a sterile brush and sieved through a sterile sieve [23, 24]. After collection, samples were frozen at − 20 °C for chemical analyses and at − 80 °C for molecular assays. Before harvesting, plants were collected from a 1 m^2^ area and the following measurements were taken: plant height (30 plants), number of shoots (30 plants), root weight (30 plants), and seed yield (30 plants).

### Physicochemical Analysis of Rhizosphere Soil

Rhizosphere soil samples for pH, total carbon (TC), total nitrogen (TN), and available phosphorus (AP) determinations were air-dried and passed through a 2 mm sieve. In contrast, N-NO_3_ and N-NH_4_ were measured in fresh soil mass. Parameters were determined by the following methods: pH in 1 M KCl by PN-EN ISO 10390:2022–09 [25], TC by PN-R-04024:1997 (Polish standard) [26], and total TN by ISO 13878:1998[27]. The concentrations of N-NH_4_ were assessed by the Continuous Flow Analysis (CFA) with spectrophotometric detection. AP was measured according to PN-R-04023:1996 [28].

### 16S rRNA Sequencing and Statistical and Bioinformatic Analyses

Soil DNA was extracted using the Magnetic Soil and Stool DNA Kit (Tiangen, China). PCR amplification of target regions was conducted using specific primers for the V3-V4 region: 314F (5′-CCTACGGNGGCWGCAG-3′) and 785R (5′-GACTACHVGGTATCTAATCC-3′). The PCR reactions were performed using 15 μL of Phusion® High—Fidelity PCR Master Mix, 0.2 μM of forward and reverse primers, and approximately 10 ng of template DNA. The thermocycling conditions were as follows: initial denaturation at 98 °C for 1 min, followed by 30 cycles of denaturation at 98 °C for 10 s, annealing at 50 °C for 30 s, and elongation at 72 °C for 30 s and 72 °C for 5 min. PCR products of the appropriate size were selected by electrophoresis on a 2% agarose gel. Libraries were prepared using the NEB Next® Ultra™ II FS DNA PCR-free Library Prep Kit (New England Biolabs, USA) following the manufacturer’s recommendations. Sequencing was performed on an Illumina NovaSeq 6000 System sequencer (Novogene, Germany), with paired-end reads generating 250 bp raw reads. The raw data were filtered and de-duplicated using the DADA2 method [29, 30]. De-duplicated sequences, after noise reduction with DADA2, were termed Amplicon Sequence Variants (ASVs) or feature sequences (corresponding to OTU representative sequences). Taxonomic annotation was carried out using the Naive Bayes classifier [31, 32] with the Silva 138.1 database. Based on the taxonomic annotation results, the top taxa from the phylum (top 6) and genus (top 8) levels were selected to create a histogram of the distribution of relative taxon abundance.

### Alpha and Beta Diversity

To analyze the diversity and richness in the sample, alpha diversity was calculated from 4 indices in QIIME2, including Observed_features, Chao1, Shannon, and Simpson.

Principal component analysis (PCA) and principal coordinate analysis (PCoA) (using weighted unifrac) were carried out using the ade4 package and the ggplot2 package with R software (version 4.0.3).

### Statistical Analysis

LEfSe, *t*-test, and MetaStat analyses were conducted to identify significant differences in the bacterial community at the phylum and genus levels. These analyses were performed using the vegan and ggplot2 packages within R.

Tukey’s HSD (honest significant difference) test (with a significance level of *p* = 0.05) and Spearman correlation were performed to assess correlations between dominant taxa at the phylum and genus levels and chemical parameters. The results were processed statistically using Statistica 6.0.

## Results

### Plant Growth-Promoting Traits and Identification of *Pseudomonas* sp. KR227 and *Azotobacter* sp. PBC1

The isolates selected for further analysis were chosen based on specific criteria that were deemed essential for the study. The study focuses on one nitrogen-fixing isolate (diazotroph) chosen for its ability to produce IAA to promote root growth and to solubilize inaccessible forms of phosphorus, thereby making it available to plants. Following the screening process, two isolates were selected and identified via 16S rRNA gene sequencing as *Pseudomonas* sp. KR227 and *Azotobacter* sp. PBC1. Importantly, the isolates did not exhibit antagonistic interactions with each other. *Azotobacter* sp. PBC1 was capable of fixing atmospheric nitrogen. Both bacterial strains produced IAA on LB medium supplemented with tryptophan, with *Pseudomonas* sp. KR227 showing the strongest phosphorus solubilization ability among the isolates. However, neither isolate demonstrated zinc solubilization nor cellulolytic activity (CMCase) (Table 1).

**Table 1:** PGP traits of bacterial strains

Strain	*Pseudomonas* sp. KR227	*Azotobacter* sp. PBC1
IAA	22.58 µg mL^−1^	41.15 µg mL^−1^
Nitrogen fixation	-	+
P solubilization	+	-
Zn solubilization	-	-
CMCase	-	-

### Plant Growth Traits

An increase in the values of parameters such as shoot weight, root length, root weight, and seed yield was observed following the application of the P2A consortium. However, statistical analysis indicated that these differences were not significant (Table 2). Nevertheless, after inoculation, two parameters showed substantial enhancement: root weight increased by 21.95% and seed yield by 18.94% compared to the control (Table 2).

**Table 2:** Plant growth traits after application of P2A

Treatment	Shoot length (cm)	Shoot weight (g)	Root length (cm)	Root weight (g)	Seed yield (g)
C.R	157.57a	1573.67a	18.67a	101.23a	415.75a
P2A.R	157.72a	1647.33a	19.63a	123.45a	494.5a

Means in columns with the same letter do not differ significantly at *p* < 0.05 in Tukey’s HSD test.

### Physicochemical Properties of Rhizosphere

P2A inoculation led to several statistically significant changes in the physicochemical properties of the rhizosphere soil (Table 2). The pH levels at each time point were higher in the inoculated soil (5.03–5.17) compared to the control (4.77–4.87). No significant differences were observed in nitrate levels. However, for ammonium, a significant increase of 35.76% was noted in the inoculated rhizosphere at the first time point (8.73 mg kg^−1^) compared to the control (6.43 mg kg^−1^), with no significant differences at subsequent time points. Significant differences were also observed in available phosphorus, where the P2A-inoculated soil showed a 35.05% increase at the first time point compared to the control. In contrast, no significant differences were found in TN and TOC (Table 3).

**Table 3:** Physicochemical properties of rhizosphere soil and alpha diversity of oilseed rape rhizobacterial community in three time points

	1st time point		2nd time point		3rd time point	
Treatment	C.R	P2A.R	C.R.2	P2A.R.2	C.R.3	P2A.R.3
pH	4.87a	5.1 a	4.77a	5.03b	4.83a	5.13b
N-NO_3_ (mg kg^−1^)	6.17a	6.73a	4.57a	4.73a	4.57a	4.73a
N-NH_4_ (mg kg^−1^)	6.43a	8.73b	11.9a	12.4a	7.83a	8.53a
AP (mg kg^−1^)	9.87a	13.33b	9.43a	9.87a	9.93a	12.53a
TN (%)	0.13a	0.13a	0.14a	0.14a	0.12a	0.13a
TOC (%)	1.4a	1.42a	1.47a	1.5a	1.44a	1.44a
Chao1	2902.6a	2817.5a	1649.8a	1612.4a	2067.0a	2147.9a
Observed features	2830a	2750a	1644a	1594a	2000a	2100a
Shannon	10.135a	9.868a	9.285a	8.763a	9.663a	9.652a
Simpson	0.995a	0.993a	0.992a	0.984a	0.993a	0.992a

*C.R*, control rhizosphere soil; *P2A.R*, rhizosphere inoculated with P2A; *AP*, P available (P_2_O_5_); *TOC*, total organic carbon; *TN*, total nitrogen; means in rows with the same letter do not differ significantly at *p* < 0.05 in Tukey’s HSD test

### Alpha Diversity

The bacterial community’s response to the introduction of the P2A consortium was analyzed by sequencing the V3–V4 regions of 16S rRNA genes, resulting in a total of 1,615,696 raw sequences (813,498 at the first time point, 414,922 at the second, and 387,276 at the third). Inoculation with *Pseudomonas* sp. KR227 and *Azotobacter* sp. PBC1 did not result in significant differences in alpha diversity. Throughout the study, individual parameter values remained within the following ranges: Chao1 index (1612.4–2902.6, with the highest values recorded at the first time point), observed features (1594–2830), Shannon index (8.763–10.135), and Simpson index (0.984–0.995) (Table 3).

### Beta Diversity

**Fig. 1:**
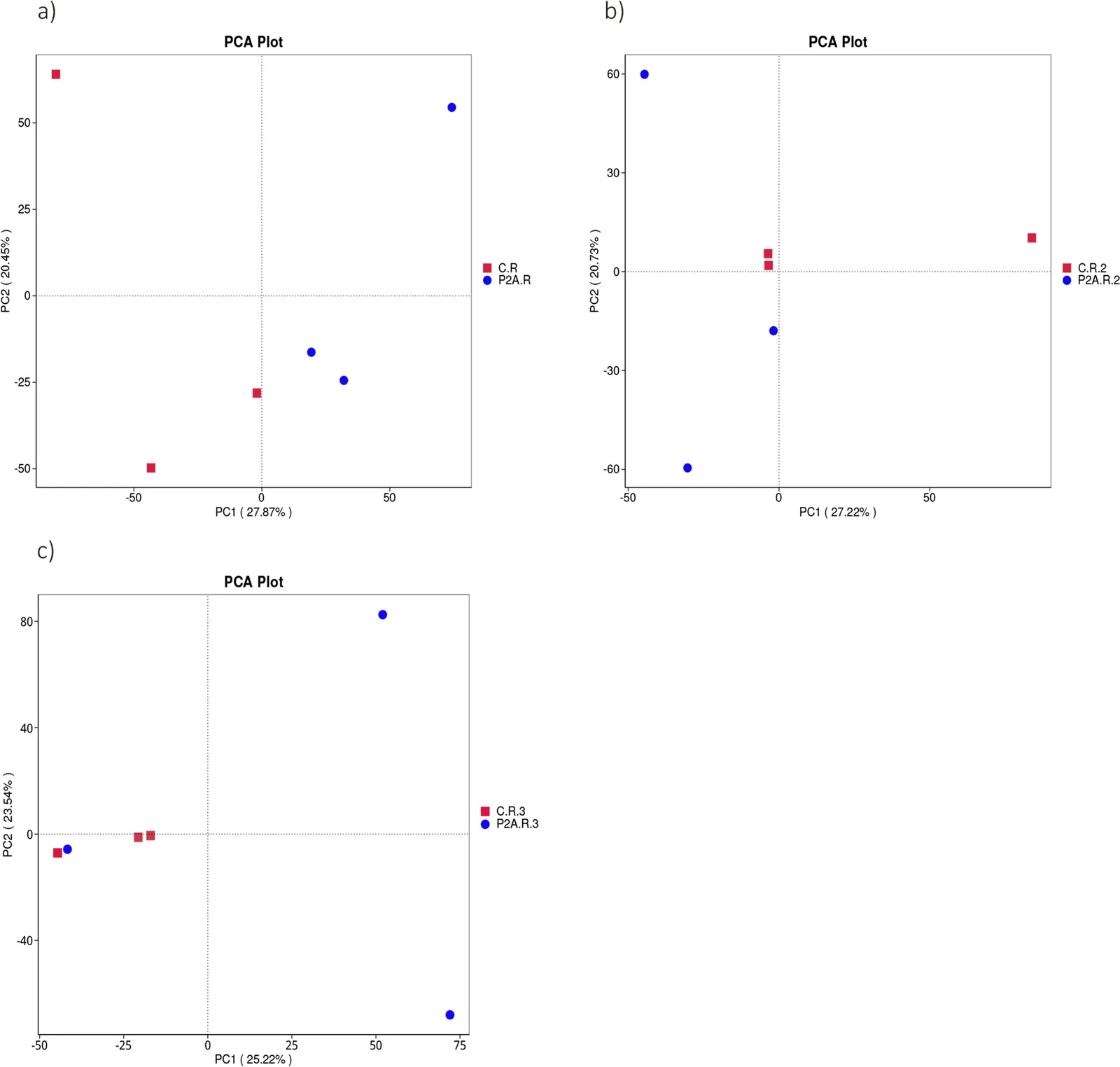
PCA of the bacterial community of rhizospheric soil in different treatments: C.R, control rhizosphere soil, P2A.R, rhizosphere inoculated with P2A; **a** first time point, **b** second time point, and **c** time point

PCA analysis showed that individual samples did not always form clusters and exhibited considerable heterogeneity. On the first sampling date, two pairs of samples from each treatment were evident, while two samples were distinctly separated from the others, a result attributed to the variance along the PC2 axis (20.45%) (Fig. 1a). At the second and third time points, only the inoculated samples formed a pair (second date) or a cluster (third date), with the P2A-inoculated samples showing clear differences from one another (Fig. 1b and c). Similar patterns were observed in the PCoA analysis using the weighted-unifrac algorithm (Supplement, Fig. 1a, b, c).

### Bacterial Community

**Fig. 2:**
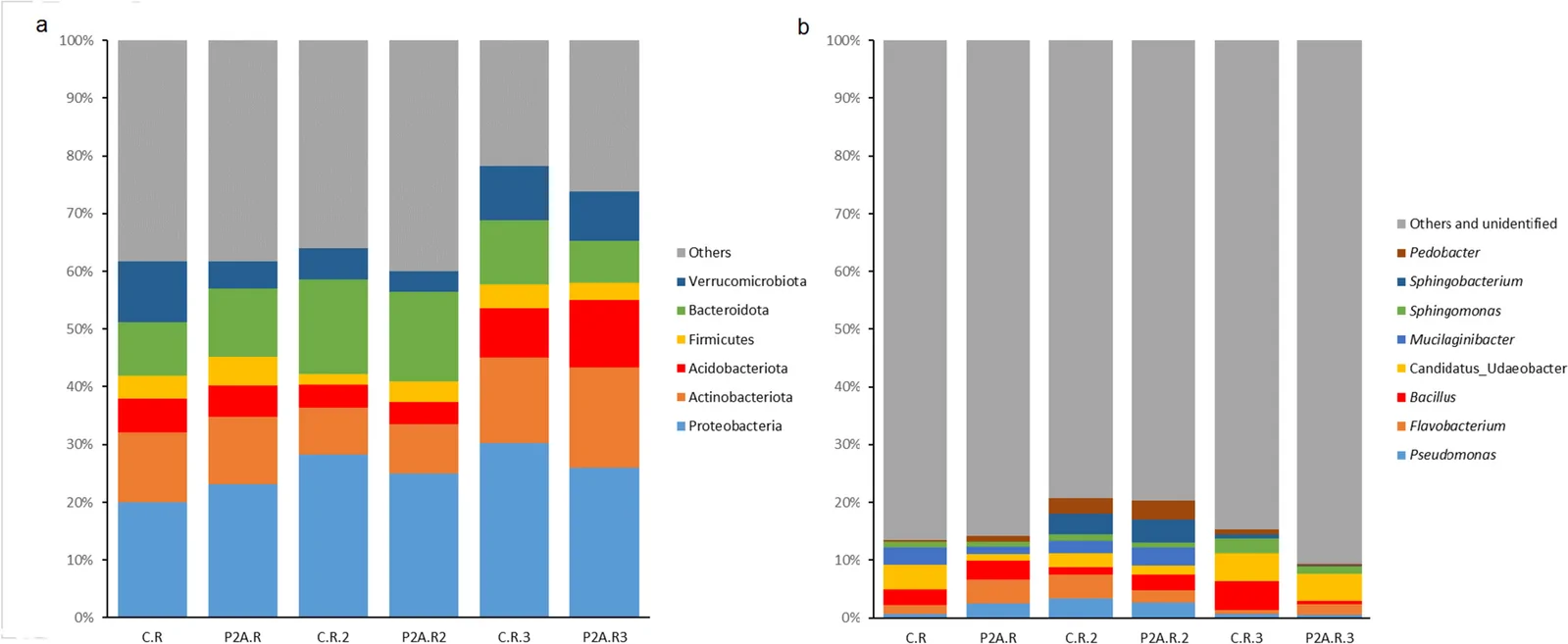
Relative abundance of the dominant bacterial phyla (**a**) and genera (**b**) in different treatments: C.R, control rhizosphere soil; P2A.R, rhizosphere inoculated with P2A; 1—first time point, 2—second time point, 3—third time point

In the analyzed samples, the dominant phyla included Proteobacteria (19.95–30.26%), Actinobacteriota (8.15–17.25%), Acidobacteriota (3.78%–11.68%), Bacteroidota (7.29–16.39%), Firmicutes (1.89–5.00%), and Verrucomicrobiota (3.58–10.5%) (Fig. 2a).

**Fig. 3:**
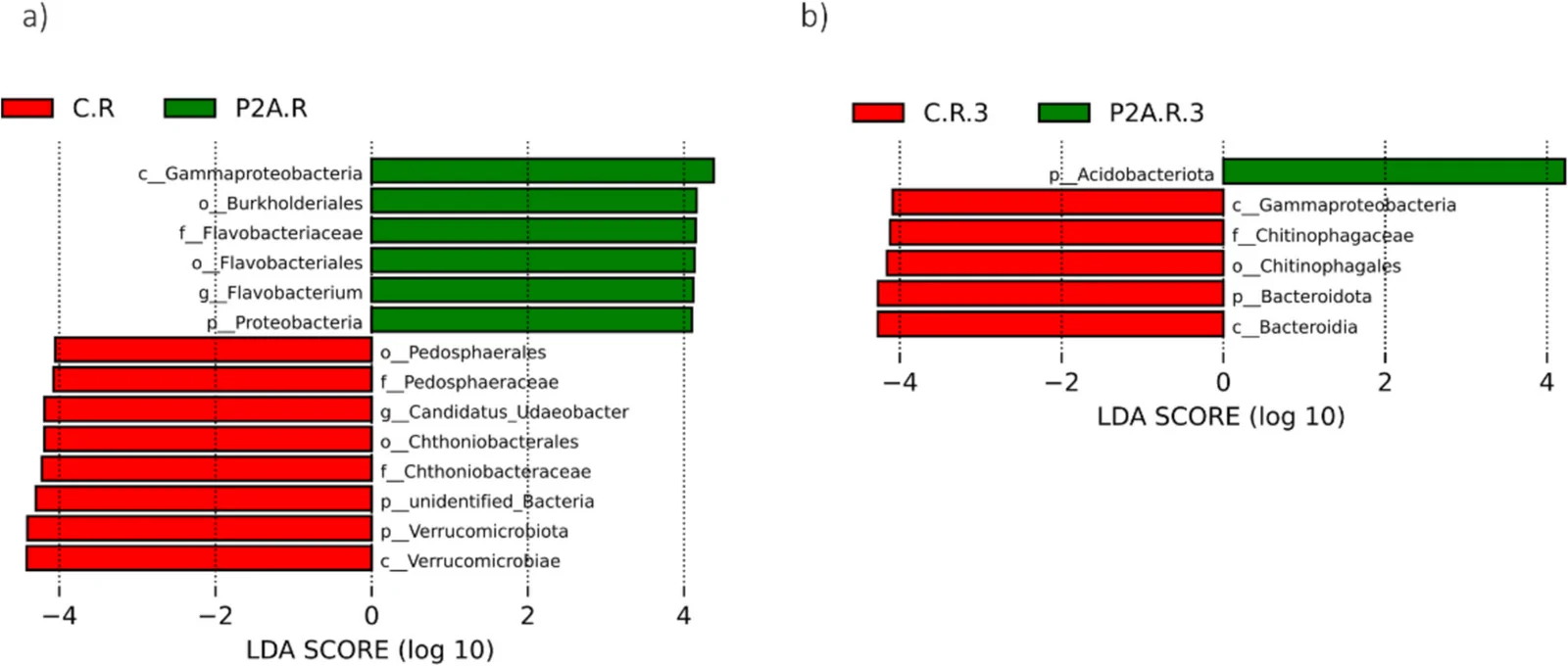
LEfSe analysis of the rhizosphere bacterial community between the following treatments: C.R, control rhizosphere soil; P2A.R, rhizosphere inoculated with P2A; **a** first time point, **b** third time point (only first time point and third time point; there were no significant differences at the second time point)

**Fig. 4:**
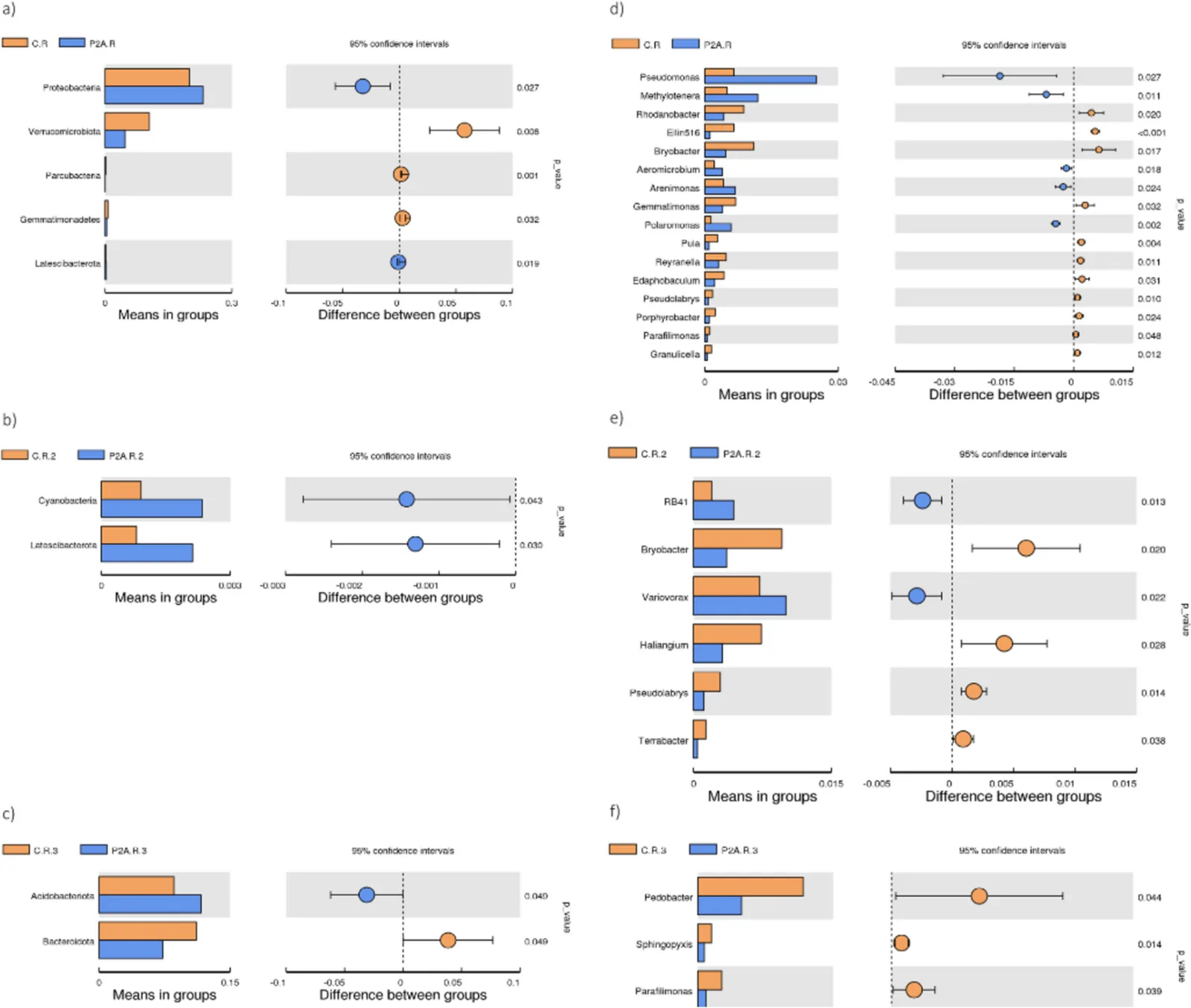
*T*-test analysis of the rhizosphere bacterial community between the following treatments: C.R, control rhizosphere soil; P2A.R, rhizosphere inoculated with P2A; phyla—**a, b, c** (three time points); genera—**d, e, f** (three time points)

Three weeks post-inoculation, the relative abundance of Proteobacteria increased (Fig. 2a), while Verrucomicrobiota decreased. These findings were confirmed by LEfSe (Fig. 3a), *t*-test (Fig. 4a), and MetaStat (Supplement – Fig. 2a). In contrast, no significant differences were observed in the dominant bacterial phyla at the BBCH 75 growth stage (second time point). However, a significant increase in Cyanobacteria and Latescibacterota was detected in the P2A-inoculated rhizosphere, as shown by the *t*-test and MetaStat (Fig. 4b; supplement – Fig. 2). Before the harvest, LEfSe (Fig. 3b), *t*-test (Fig. 4c), and MetaStat (Supplementary Fig. 2a) indicated an increase in Acidobacteriota and a decrease in Bacteroidota in the inoculated soil. In contrast, the Tukey test showed a decrease in Firmicutes in non-inoculated soil (Supplement – Table 1).

At the genus level, dominant taxa in most samples included *Candidatus* Udaebacter (1.09–4.85%), *Bacillus* (0.57–3.39), *Pseudomonas* (0.45–3.37), and *Flavobacterium* (0.67–4.14) (Fig. 2b). For genus-level sequences, the *t*-test (Fig. 4d) and Metastat (Supplement – Fig. 2b) documented an increase in the population of *Pseudomona*s in the inoculated rhizosphere at the first time point, suggesting that *Pseudomonas* sp. KR227 persisted in the soil in greater abundance for at least 3 weeks. Conversely, after 3 weeks, LEfSe and Tukey tests (also *t*-test and MetaStat) showed a significant decrease in population of *Candidatus* Udaeobacter in the inoculated soil compared to control (Fig. 3a, supplement – Table 2). Additionally, the *t*-test (Fig. 4d) and MetaStat (Supplement – Fig. 2b) revealed higher abundance of the genus *Methylotenera* in the inoculated soil, while *Rhodanobacte*r and *Ellin516* were less abundant in the inoculated soil compared to the control. No significant differences were found in the dominant genera at the next time point (Fig. 4e; supplement – Table 2). However, significant differences were detected in taxa such as *RB41*, *Bryobacter*, *Variovorax*, and *Haliangium* at the second time point (Fig. 4e, supplement – Fig. 2). At the third time point, although no significant changes in the dominant populations were observed, the *t*-test (Fig. 4f) and MetaStat (Supplement—Fig. 2b) indicated significant decreases in bacteria from genera such as *Pedobacter*, *Sphingopyxis*, and *Parafilimonas* in the inoculated rhizosphere. Overall, these changes were not consistent and did not persist throughout the growing season.

### Relationship Between the Bacterial Community and the Physicochemical Properties of the Soil

Spearman correlation analysis was conducted to examine the relationship between dominant bacterial taxa and physicochemical properties of the soil (Table 4). At the phylum level, the analysis showed that Proteobacteria (*p* < 0.05) (first time point), Verrucomicrobiota (second time point), and Acidobacteriota (third time point) were positively correlated with available P (*p* < 0.05). Besides, positive correlations were observed between Verrucomicrobiota and N-NH4 (first time point) (*p* < 0.05) and between Firmicutes (third time point) and N-NH4 (*p* < 0.05). However, negative correlations were found between Actinobacteriota (second time point) and both N-NO_3_ and N-NH_4_, as well as between Verrucomicrobiota (third time point) and N-NO_3_ (*p* < 0.05). Moreover, Bacteroidota were negatively correlated with pH (*p* < 0.05).

**Table 4:** Spearman’s correlation between taxa and physicochemical properties

Time points	Taxa	pH	N-NO_3_	N-NH_4_	P available (P_2_O_5_)	TOC	TN
1st time point	p-Proteobacteria	0.52	0.12	− 0.77	0.83*	− 0.70	0.34
	p-Actinobacteriota	− 0.06	0.49	− 0.09	− 0.49	0.12	− 0.34
	p-Acidobacteriota	− 0.09	0.23	− 0.09	− 0.37	0.58	− 0.51
	p-Firmicutes	0.64	− 0.03	− 0.31	0.77	− 0.55	0.51
	p-Bacteroidota	0.75	0.64	− 0.66	0.49	− 0.29	− 0.51
	p-Verrucomicrobiota	− 0.75	− 0.64	0.94*	− 0.66	0.64	0.17
2nd time point	p-Proteobacteria	− 0.26	0.37	0.17	0.14	0.26	0.41
	p-Actinobacteriota	0.26	− 0.83*	− 0.81*	− 0.71	− 0.2	− 0.62
	p-Acidobacteriota	− 0.44	− 0.71	− 0.64	− 0.37	− 0.26	− 0.41
	p-Firmicutes	0.79	0.14	− 0.23	− 0.49	0.09	0.00
	p-Bacteroidota	0.26	0.6	0.64	0.43	0.09	0.41
	p-Verrucomicrobiota	− 0.26	− 0.03	0.64	0.83*	0.37	− 0.41
3rd time point	p-Proteobacteria	− 0.62	− 0.14	− 0.14	− 0.66	− 0.09	− 0.1
	p-Actinobacteriota	0.79	0.37	0.03	0.20	− 0.54	0.29
	p-Acidobacteriota	0.79	0.09	0.03	0.83*	0.14	0.29
	p-Firmicutes	− 0.44	0.26	0.94*	− 0.14	0.49	0.49
	p-Bacteroidota	− 0.88*	− 0.26	− 0.09	− 0.77	− 0.03	− 0.49
	p-Verrucomicrobiota	− 0.09	− 0.83*	0.31	0.26	0.60	0.10
1st time point	g-*Flavobacterium*	0.78	0.12	− 0.6	0.83*	0.00	− 0.17
	g-*Pseudomonas*	0.61	− 0.03	− 0.6	0.89*	− 0.35	0.17
	g-*Bacillus*	0.41	− 0.12	− 0.2	0.6	− 0.61	0.68
	g-*Mucilaginibacter*	− 0.64	0.06	0.14	− 0.54	− 0.49	0.34
	g-*Candidatus *Udaeobacter	− 0.90*	− 0.55	0.83*	− 0.77	0.52	0.17
	g-*Sphingomonas*	− 0.52	0.46	0.09	− 0.83*	0.03	− 0.51
	g-*Pedobacter*	0.46	0.06	− 0.71	0.77	− 0.46	0.17
2nd time point	g-*Flavobacterium*	− 0.53	0.37	0.84*	0.94*	0.03	0.21
	g-*Pseudomonas*	0.18	0.37	0.49	0.37	0.14	0.21
	g-*Bacillus*	0.79	0.03	− 0.06	− 0.26	0.03	− 0.21
	g-*Mucilaginibacter*	0.18	− 0.43	0.14	0.26	− 0.2	− 0.62
	g-*Candidatus *Udaeobacter	− 0.09	− 0.26	0.58	0.77	0.14	− 0.62
	g-*Sphingomonas*	− 0.62	− 0.71	− 0.17	0.2	− 0.09	− 0.62
	g-*Pedobacter*	0.44	0.49	0.41	0.14	− 0.09	0.41
	g-*Sphingobacterium*	0.44	0.49	0.41	0.14	− 0.09	0.41
3rd time point	g-*Flavobacterium*	− 0.18	− 0.94*	− 0.09	0.14	0.37	− 0.49
	g-*Pseudomonas*	− 0.79	0.37	− 0.09	− 0.71	− 0.2	− 0.49
	g-*Bacillus*	− 0.44	− 0.03	0.89*	− 0.09	0.6	0.49
	g-*Candidatus *Udaeobacter	− 0.35	− 0.77	0.26	0.09	0.66	0.10
	g-*Sphingomonas*	− 0.71	0.03	− 0.09	− 0.94*	− 0.43	− 0.49
	g-*Pedobacter*	− 0.88*	0.20	0.26	− 0.77	− 0.09	− 0.49
	g-*Sphingobacterium*	− 0.79	− 0.09	0.03	− 0.26	0.43	− 0.29

*TOC*, total organic carbon; *TN*, total nitrogen. *Marked correlations are significant at *p* < 0.05

At the genus level, the Spearman analysis revealed more correlations than at the phylum level. For instance, *Flavobacterium* was positively correlated with AP both at the first and second time points and with N-NH_4_ at the second time point while showing a negative correlation with N-NO_3_ at the third time point. Also, *Bacillus* spp. were positively correlated with N-NH_4_ at the third time point (*p* < 0.05). In turn, *Pseudomona*s showed a positive correlation with AP at the first time point (*p* < 0.05). In addition, bacteria of the genera *Candidatus* Udaeobacter (first time point) and *Pedobacter* (third time point*)* were negatively correlated with pH values. Interestingly, *Candidatus* Udaeobacter also showed a positive correlation with N-NH_4_ at the first time point. Finally, *Sphinogomonas* exhibited negative correlations with AP at both the first and third time points (*p* < 0.05).

## Discussion

Plant growth-promoting bacteria (PGPB) are among the most effective solutions for reducing the need for mineral fertilization [33]. To date, only a few studies have examined the application of growth-promoting bacteria for oilseed rape [16, 18, 34], and even fewer have done so under field conditions [16, 35]. Therefore, this study concerns the impact of a selected PGPB consortium, consisting of *Pseudomonas* sp. KR227 and *Azotobacter* sp. PBC1, on the growth characteristics of winter oilseed rape, particularly on the native bacterial community of the rhizosphere in field conditions. The study found that the bacterial consortium, characterized by IAA production, nitrogen fixation, and phosphorus solubilization, contributed to a slight increase in most of the studied parameters of plant growth (root mass, shoot yield, and seed yield). These effects were reflected in the rhizosphere’s chemical properties, particularly in the increase in plant-available ammonium and phosphorus during the study period, with a statistically significant increase observed after 3 weeks. Moreover, the increase in root mass may be linked to elevated IAA levels produced by both strains. Previous studies have reported similar growth-promotion effects of beneficial bacteria on oilseed rape. For instance, Swiontek Brzezinska et al. [34] used a microbial consortium (*Pseudomonas* sp. B14, *Sphingobacterium* sp. B16, and *Microbacterium* sp. B19) and observed an increase in leaf count and shoot length in a 6-week experiment under controlled conditions (growth chamber). Other studies using *Bacillus* spp. have reported enhanced root growth and seedling mass within 14 days [36], while *Bacillus* sp. Kz5 and *Enterobacter* sp. Kz15 significantly increased plant biomass in a 21-day greenhouse experiment [37]. However, under field conditions, Schmidt et al. [38] found that a bacterial consortium (*Achromobacter piechaudii* AP_RD9, *Pseudomonas* sp. PCh_RH1, *Pseudomonas* sp. PG_RD39 (*P. fluorescens* clade), and *Stenotrophomonas* sp. SS_RD24) did not yield significant growth-promoting effects for rapeseed.

The rhizobacterial community is a primary factor influencing biochemical processes in rhizosphere soil [39]. Therefore, as previously mentioned, the main objective of this study is to investigate the response of the rhizobacterial community to the introduction of a plant growth-promoting bacterial consortium. To the best of our knowledge, this is the only study to describe the effect of a consortium containing bacterial strains of the genera *Pseudomonas* and *Azotobacter* on the native bacterial community in an experiment conducted under field conditions.

Following the introduction of the tested consortium P2A (*Pseudomonas* sp. KR227 and *Azotobacter* PBC1), the alpha diversity of the rapeseed rhizobacteria did not change significantly at any time point, which can generally be considered a favorable result from an application perspective. Similarly, following the introduction of *Pseudomonas brassicacearum* CDVBN10, no significant changes in the alpha diversity of the bacteria in oilseed rape roots were observed [17]. Additionally, Chen et al. [40] reported no significant changes in the alpha diversity of the wheat rhizosphere bacterial community following the application of PGPB.

PCA analysis showed that the individual P2A inoculated samples were slightly more heterogeneous than the uninoculated samples, as indicated by the pair/cluster formation of the control samples, especially at the second and third time points. The greater heterogeneity observed in the inoculated samples may be attributed to soil variability within the study area. More specifically, the consortium, influenced by subtle differences in soil conditions, may have modulated the oilseed rape rhizobacterial community differently. Similar observations have been reported by Liu et al. [18] and Khanghahi et al. [41].

As previously documented, the dominant bacterial communities in the oilseed rape rhizosphere include the phyla Proteobacteria, Actinobacteriota, Acidobacteria, Verrucomicrobiota, Chloroflexi, and Firmicutes; these patterns are largely consistent with our findings [18, 42–44]. The bacterial community at the phylum level showed temporal changes after P2A application, though none of these changes persisted through subsequent plant growth phases. Three weeks after application (plant phase – BBCH 65), the abundance of Proteobacteria increased, while the number of Verrucomicrobiota decreased. Proteobacteria are generally fast-growing copiotrophs [45], and an increase in N-NH_4_ content in inoculated soil (three weeks post-application) may have contributed to their rise in abundance. A similar increase in Proteobacteria following PGPB application has been reported by other authors [18]. Liu et al. [18] observed a slight increase in the phylum Proteobacteria in the rhizobacterial community 17 days after applying *Stenotrophomonas rhizophila* DSM 14405 T. However, similarly to the P2A application, this change did not persist, with a decline in this phylum recorded at later time points (23 and 59 days post-application) in their greenhouse experiment. Notably, they also observed a significant increase in Proteobacteria 59 days after applying *Bacillus amyloliquefaciens* FZB42 [18]. On the other hand, no changes in the abundance of the phylum Proteobacteria and Verrucomicrobiota were reported by Jiménez-Gómez [17] after applying *P. brassicacearum* CDVBN10 (endophyte) to the root microbiome of oilseed rape, though their study only focused on the mature stage of the plant. In turn, the decline in Verrucomicrobiota may be related to their oligotrophic lifestyle [46–48]. However, recent studies increasingly suggest that some members of the Verrucomicrobiota may also exhibit copiotrophic behavior [49–51].

At the second time point (plant phase – BBCH 75), no changes in the dominant phyla of the native rhizobacterial community were recorded. However, at the mature phase, an increase in Acidobacteriota and a decrease in Bacteroidota and Firmicutes (as indicated by the Tukey test) were observed in the inoculated soil. A similar decrease in Bacteroidota and an increase in Acidobacteriota (after 23 and 59 days) were also reported Liu et al. [18]. The decline in Bacteroidota may be associated with its copiotrophic lifestyle [45], suggesting that a slight decrease in nutrient availability could have influenced this outcome. Meanwhile, the increase in Acidobacteria may be positively correlated with AP, though the overall relationship remains unclear. In contrast, other studies have reported different patterns. For instance, Kröber et al. [52] observed a significant decrease in relative abundance of the phylum Proteobacteria after the application of *B. amyloliquefaciens* FZB42 to lettuce under field conditions. It is important to note that the differences between the studies depend not only on the plant or the PGPB used but also on soil properties—particularly in such a dynamic environment as the rhizosphere—and on climatic conditions.

The occurrence of dominant genera is partially consistent with previous reports on the bacterial community of the oilseed rape rhizosphere [18, 53, 54]. For example, the genus *Flavobacterium* has also been reported as dominant in the rhizosphere of oilseed rape [54].

The applied consortium significantly affected dominant genera only at the first time point. P2A increased the abundance of *Pseudomona*s, which may be due to the introduction of *Paenibacillus* sp. KR227 into the soil. This phenomenon is likely related to the increased content of available phosphorus in the soil, which may have resulted from the activity of this strain. The consortium was also shown to increase the abundance of *Flavobacterium* and decrease the abundance of *Candidatus* Udaebacter. The enhancement of the *Flavobacterium* population, as shown by the Spearman test, can be explained by the increased phosphorus content. Overall, the rise in the abundance of *Pseudomonas* and *Flavobacterium* in the rhizosphere appears beneficial to plants, as both genera contain plant growth-promoting bacteria [55–57]. Notably, *Pseudomonas* is rich in PGPB, and many species within this genus are known for producing phytohormones and ACC deaminase (alleviate drought stress), as well as for their ability to solubilize phosphorus [55, 58]. On the other hand, the observed decline in the abundance of the antibiotic-resistant genus *Candidatus* Udaebacter, which plays a role in the hydrogen cycle, may be slightly unfavorable [59]. However, it is important to note that these shifts in the oilseed rape rhizobacterial community at genus level were not permanent. A slightly different outcome was reported by Liu et al. [18], who found that the introduction of *Rhodobacter sphaeroides* EBL0706 and *B. amyloliquefaciens* FZB42 (introduced separately) increased the abundance of the family Pseudomonadaceae, which includes *Pseudomona*s spp., 59 days after inoculation compared to the control. Additionally, *R. sphaeroides* EBL0706 increased the population of the family Flavobacteriaceae 59 days post-inoculation, although the highest abundance of this family was observed in uninoculated soil 23 days after PGPB application [18].

## Conclusions

A consortium comprising *Pseudomonas* and *Azotobacter* somewhat promoted the growth of winter oilseed rape and significantly shifted the abundance of certain taxa at different time points, although did not affect the diversity of rhizosphere bacteria. The growth-promoting effects were likely related to the biochemical characteristics of the consortium, specifically IAA production, phosphorus solubilization, and atmospheric nitrogen fixation. These mechanisms contributed to a significant increase in available phosphorus (AP) and ammonium nitrogen (N-NH4) in the rhizosphere three weeks after inoculation. Therefore, the tested P2A consortium shows potential as a biostimulant for rapeseed cultivation. However, further research is necessary to evaluate the effects of the P2A consortium on the growth of other plants and the functional microbiota, ideally using RNA-seq to gain deeper insights.

## Supplementary Information

Below is the link to the electronic supplementary material.

Supplementary file1 (RAR 749 KB)

Supplementary file2 (DOCX 15.2 KB)

## Data Availability

Sequencing data has been deposited in Sequence Read Archive (SRA) data (NCBI) under the following accession number: PRJNA1092498.
