# Supplementary material for: Non-native PGPB Consortium Altered the Rhizobacterial Community and Slightly Stimulated the Growth of Winter Oilseed Rape (Brassica napus L.) Under Field Conditions
Source: Microb Ecol. 2025 Jan 8;87:168. doi: 10.1007/s00248-024-02471-3 (PMC11711131; doi:10.1007/s00248-024-02471-3)
Supplement: Supplementary file 1 [file 248_2024_2471_MOESM2_ESM.docx]

Non-native PGPB consortium altered the rhizobacterial community and slightly stimulated the growth of winter oilseed rape (*Brassica napus* L.) under filed conditions

Dobrzyński J.*, Kulkova I. Jakubowska Z., Wróbel B.

Institute of Technology and Life Sciences—National Research Institute, Falenty, 3 Hrabska Avenue, 05-090 Raszyn, Poland

*Corresponding author: e-mail: [j.dobrzynski@itp.edu.pl](mailto:j.dobrzynski@itp.edu.pl)

**Supplement, table 1.** Relative abundances of rhizobacterial community at phylum level

| Taxa | CR | P2A |
| --- | --- | --- |
| First time point | | |
| Firmicutes | 0.0410a | 0.0500a |
| Proteobacteria | 0.1995b | 0.2318a |
| Bacteroidota | 0.0926a | 0.1175a |
| Verrucomicrobiota | 0.1050a | 0.0475b |
| Acidobacteriota | 0.0573a | 0.0534a |
| Actinobacteriota | 0.1219a | 0.1168a |
| Second time point | | |
| Proteobacteria | 0.2821a | 0.2498a |
| Actinobacteriota | 0.0815a | 0.0857a |
| Acidobacteriota | 0.1639a | 0.1539a |
| Bacteroidota | 0.0189a | 0.0365a |
| Firmicutes | 0.0397a | 0.0379a |
| Verrucomicrobiota | 0.0540a | 0.0359a |
| Third time point | | |
| Proteobacteria | 0.3026a | 0.2604a |
| Actinobacteria | 0.1481a | 0.1725a |
| Firmicutes | 0.1115a | 0.0730b |
| Bacteroidota | 0.0932a | 0.0846a |
| Acidobacteriota | 0.0178a | 0.0320a |
| Verrucomicrobiota | 0.0412a | 0.0302a |
|  |  |  |

Means in columns with the same letter do not differ significantly at p < 0.05 in Tukey’s HSD test.

**Supplement, table 2.** Relative abundances of rhizobacterial community at genus level

| Taxa | CR | P2A |
| --- | --- | --- |
| First time point | | |
| *Flavobacterium* | 0.0154b | 0.0414a |
| *Pseudomonas* | 0.0065b | 0.0251a |
| *Bacillus* | 0.0283a | 0.0339a |
| *Mucilaginibacter* | 0.0302a | 0.0135a |
| *Candidatus_*Udaeobacter | 0.0420a | 0.0109b |
| Second time point | | |
| *Pseudomonas* | 0.0337a | 0.0260a |
| *Sphingobacterium* | 0.0362a | 0.0402a |
| *Sphingomonas* | 0.0121a | 0.0092a |
| *Bacillus* | 0.0139a | 0.0268a |
| *Candidatus_*Udaeobacter | 0.0239a | 0.0163a |
| *Flavobacterium* | 0.0414a | 0.0223a |
| Third time point | | |
| *Pseudomonas* | 0.0072a | 0.0045a |
| *Candidatus_Udaeobacter* | 0.0485a | 0.0468a |
| *Bacillus* | 0.0311a | 0.0188a |
| *Sphingomonas* | 0.0244a | 0.0133a |

Means in columns with the same letter do not differ significantly at p < 0.05 in Tukey’s HSD test.
